# Labor and birth care practices associated with exclusive breastfeeding at discharge

**DOI:** 10.1590/0034-7167-2025-0305

**Published:** 2026-09-21

**Authors:** Manuela Beatriz Velho, Isabel Cristina Alves Maliska, Zannis Benevides de Andrade, Franciane Zabloski Vieira, Luana Costa Albino, Thayná Ventura, Liliana Maria Ferreira Vicente, Roxana Knobel

**Affiliations:** I Universidade Federal de Santa Catarina. Florianópolis, Santa Catarina, Brazil

**Keywords:** Labor, Obstetric, Obstetric Care, Professional Practice, Breast Feeding, Health Policy., Trabajo de Parto, Atención Obstétrica, Práctica Professional, Lactancia Materna, Política de Salud.

## Abstract

**Objectives:**

to identify the prevalence of labor and birth care practices and their association with exclusive breastfeeding at discharge.

**Methods:**

cross-sectional study based on the Woman-Friendly Care criterion, conducted with 756 postpartum women at a Baby-Friendly Hospital. Proportions and 95% CIs were calculated, and the chi-square test was used.

**Results:**

the prevalence of exclusive breastfeeding at discharge was 85.0%. Good practices associated with a higher prevalence of the outcome included privacy and a welcoming environment (73.9%), skin-to-skin contact (83.2%), and assistance with positioning the baby to breastfeed (78.6%). Among invasive procedures, cesarean birth (36.0%) was associated with a lower prevalence of the outcome.

**Conclusions:**

labor and birth care practices were associated with exclusive breastfeeding at discharge, highlighting the importance of public policies that ensure respectful, woman-centered, evidence-based care.

## RESUMEN

**Objetivos:**

identificar la prevalencia de prácticas de atención al parto y nacimiento y su relación con la lactancia materna exclusiva al alta.

**Métodos:**

estudio transversal, basado en el Cuidado Amigo de la Mujer, con 756 puérperas de un Hospital Amigo del Niño. Se calcularon proporciones, intervalos de confianza del 95% y la prueba de chi-cuadrado.

**Resultados:**

la prevalencia de lactancia materna exclusiva al alta fue del 85,0%. Se observó mayor prevalencia y asociación del desenlace con privacidad y ambiente acogedor y con prácticas favorables a la lactancia, como contacto piel con piel y ayuda para poner al bebé al pecho. La cesárea se asoció con menor prevalencia del desenlace.

**Conclusiones:**

las prácticas de atención al parto y al nacimiento y el apoyo a la lactancia en el posparto inmediato se asociaron con la lactancia materna exclusiva al alta y refuerzan la importancia de una atención humanizada y basada en la evidencia.

## INTRODUCTION

The Baby-Friendly Hospital Initiative (BFHI) includes the Woman-Friendly Care criterion, which was incorporated into this policy in Brazil in 2014. Referred to in other countries as “Mother Friendly Care”, it encompasses a set of care practices for women during labor and birth designed to support the physiologic progression of labor. These practices include ensuring a companion of her choice; offering fluids and light foods during labor; encouraging freedom of movement during labor and allowing women to adopt their preferred birth position; providing an appropriate, calm, welcoming environment with privacy and soft lighting; ensuring access to nonpharmacological pain relief methods; offering care that limits invasive procedures to situations in which they are required because of complications and are properly explained to the woman; and, lastly, allowing a doula to provide continuous support if she so wishes^(1)^.

To improve care for women and their families during childbirth, Brazil’s Ministry of Health published the National Guidelines for Vaginal Birth Care in 2016^(2)^; in 2018, the World Health Organization (WHO) published recommendations on intrapartum care for a positive childbirth experience^(3)^. These guidelines provide scientific evidence to guide healthcare teams in labor and birth care. Their purpose is to reduce unnecessary interventions, promote the adoption of good practices, and help lower the persistently high cesarean rates that remain a concern in Brazil^(2)^.

Thus, labor and birth care practices play an important role in breastfeeding promotion, protection, and support. In addition, high-quality care supports better health for both women and children, aligning with the third Sustainable Development Goal, which aims to ensure healthy lives and promote well-being for all at all ages^(4)^.

Given the need to ensure women’s access to these practices and to promote breastfeeding, the following research question was posed: What is the prevalence of labor and birth care practices outlined under the Woman-Friendly Care criterion, and are these practices associated with exclusive breastfeeding at discharge?

## OBJECTIVES

To identify the prevalence of labor and birth care practices and their association with exclusive breastfeeding at discharge.

## METHODS

### Ethical aspects

The study followed national and international ethical guidelines and was approved by a Research Ethics Committee under CAAE No. 36214720.0.0000.0121, with authorization from the participating institution. No external funding was received.

### Study design, period, and setting

This was a quantitative, cross-sectional study conducted online. This study is reported in accordance with the Strengthening the Reporting of Observational Studies in Epidemiology (STROBE) guidelines. The study was conducted in the maternity unit of a teaching hospital in the South Region of Brazil between July 2020 and September 2021. Since 1997, the maternity unit has been designated a Baby-Friendly Hospital and has adopted a care philosophy based on principles of humanized, interdisciplinary care. It provides obstetric emergency care, a labor and delivery unit, a gynecology unit, rooming-in, and a neonatal intensive care unit, all exclusively through the Brazilian Unified Health System (SUS). A multidisciplinary team delivers care throughout the maternity unit. The institution offers medical residency programs in obstetrics and gynecology and in pediatrics, as well as an integrated multiprofessional residency program focused on women’s and children’s health.

Within the maternity unit, the labor and delivery unit has five pre-labor beds, three of them adapted as labor, birth, and postpartum rooms, as well as one isolation bed, two operating rooms, and two postanesthesia recovery beds. The rooming-in unit has 17 inpatient beds for postpartum women. The maternity unit also has a nursing team responsible for the Breastfeeding Support Center (CIAM), which provides follow-up and reinforces breastfeeding guidance.

### Population, inclusion and exclusion criteria

The study population comprised all women who gave birth at the study maternity unit during the study period. Women were eligible if they were 18 years or older and the mother-infant dyad was admitted to rooming-in after birth. Women who did not provide a contact phone number when seen by the CIAM team during their stay in rooming-in were excluded.

Between 24 and 48 hours after discharge from the maternity unit, women were contacted via a messaging app to receive 15-20 days of remote breastfeeding support from the CIAM team. At the end of that period, they were invited to participate in the study through the same app. Data were collected using an online questionnaire sent via the messaging app. The study was conducted during the COVID-19 pandemic.

### Variables

The outcome variable investigated was exclusive breastfeeding at discharge, whereas the exposure variables were labor and birth care practices, categorized as good practices (companion of her choice in the labor and delivery unit, access to fluids and light foods, use of an exercise ball, use of a birthing stool, encouragement to use massage, prolonged showering, privacy and a welcoming environment, guidance and encouragement for the companion’s participation in care, and preferred birth position) and grouped under the variable “any guidance or good practice listed above”; invasive procedures (labor induction [use of a catheter and/or vaginal tablet and/or intravenous oxytocin], labor augmentation [intravenous oxytocin], artificial rupture of membranes performed by the healthcare professional, perineal incision [episiotomy], cesarean birth) and grouped under the variable “any invasive procedure listed above”, in addition to the variable “explanation of the reason for or need for the interventions”; and breastfeeding-support practices (skin-to-skin contact immediately after birth or within the first five minutes of life, lasting at least one hour, recognition of feeding cues, and assistance with positioning the baby to breastfeed) implemented in the labor and delivery unit.

### Data analysis

The data were entered into a spreadsheet and analyzed using Stata statistical software (version 13). All variables were categorized as nominal qualitative variables (yes or no). Data analysis included absolute frequencies, proportions, and 95% confidence intervals (95% CIs) for the practices, followed by absolute frequencies, proportions, and 95% CIs for the practices and their association with exclusive breastfeeding. For breastfeeding-support practices, associations with mode of birth (vaginal birth and cesarean birth) were also investigated. The chi-square test was used to assess differences in proportions between outcome groups, with p < 0.05 considered statistically significant.

## RESULTS

**Figure 1:**
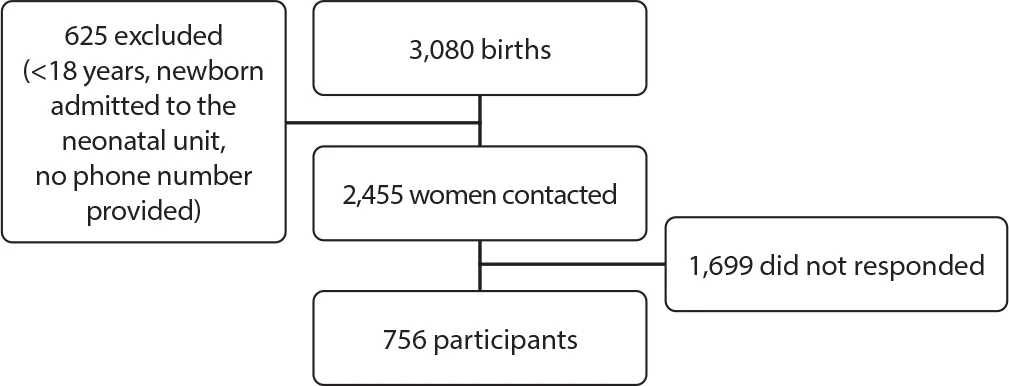
Flowchart of participant selection for the study of labor and birth care practices according to the BFHI Woman-Friendly Care criterion (n = 756), University Hospital, South Region of Brazil, 2020-2021

During the study period, 3,080 births were recorded at the institution. The 2,455 women who met the inclusion criteria were contacted via a messaging app and received the questionnaire, representing 79.7% of all women cared for in the maternity unit. Of those who received the questionnaire, 756 responded, yielding response rates of 30.8% among eligible women and 24.5% of all women cared for during the same period (Figure 1).

Among the participants, 643 were exclusively breastfeeding at discharge, representing a prevalence of 85.0%. A high prevalence of good practices was observed, with the vast majority of participants reporting access to at least one good practice or receiving guidance on good practices (97.7%). In analyses of associations with good practices, the variable “privacy and a welcoming environment” was associated with a higher prevalence of exclusive breastfeeding at discharge (87.3% vs. 78.7%) (Table 1).

**Table 1:** Good practices in labor and birth care according to the Baby-Friendly Hospital Initiative Woman-Friendly Care criterion and their association with exclusive breastfeeding at discharge (N = 756), University Hospital, South Region of Brazil, 2020-2021

	Total		Exclusive breastfeeding at discharge		
	n	% (95% CI)	n	% (95% CI)	*p* value^^*^^
**Companion of her choice**					
No	63	8.3 (6.6-10.5)	51	81.0 (71.3-90.6)	0.340
Yes	693	91.7 (89.5-93.4)	592	85.4 (82.8-88.1)	
**Access to fluids and light foods**					
No	217	28.7 (25.6-32.0)	180	83.0 (77.9-88.0)	0.303
Yes	539	71.3 (68.0-74.4)	463	85.9 (83.0-88.8)	
**Use of an exercise ball**					
No	244	32.3 (29.0-35.7)	207	84.8 (80.3-89.3)	0.908
Yes	512	67.7 (64.3-71.0)	436	85.2 (82.1-88.2)	
**Use of a birthing stool**					
No	639	84.5 (81.8-86.9)	543	85.0 (82.2-87.7)	0.891
Yes	117	15.5 (13.1-18.2)	100	85.5 (79.1-91.9)	
**Encouragement to use massage**					
No	467	61.8 (58.2-65.2)	393	84.1 (80.8-87.5)	0.378
Yes	289	38.2 (34.8-41.2)	250	86.5 (82.6-90.4)	
**Prolonged showering**					
No	243	32.1 (28.9-35.6)	204	84.0 (79.3-88.6)	0.559
Yes	513	67.9 (64.4-71.1)	439	85.6 (82.5-88.6)	
**Privacy and a welcoming environment**					
No	197	26.1 (23.0-29.3)	155	78.7 (73.0-84.4)	**0.004**
Yes	559	73.9 (70.7-77.0)	488	87.3 (84.5-90.1)	
**Guidance and encouragement for the companion’s participation in care**					
No	312	41.3 (37.8-44.8)	259	83.0 (78.8-87.2)	0.187
Yes	444	58.7 (55.2-62.2)	384	86.5 (83.3-90.0)	
**Preferred birth position^†^**					
No	93	19.2 (15.9-23.0)	78	83.9 (76.4-91.3)	0.254
Yes	391	80.8 (77.0-84.1)	345	88.2 (85.1-81.4)	
**Any guidance or good practice listed above**					
No	17	2.3 (1.4-3.6)	12	70.6 (48.9-92.2)	0.091
Yes	739	97.7 (96.4-98.6)	631	85.4 (82.8-87.9)	

* *Chi-square test; †Women who underwent cesarean birth were excluded (n = 484).*

Regarding invasive procedures, 78.7% of participants reported undergoing at least one invasive procedure, and 93.8% reported receiving an explanation of the reason for or need for the interventions. A lower prevalence of exclusive breastfeeding at discharge was observed among women who underwent cesarean birth (80.9% vs. 87.4%) and among those who underwent at least one invasive procedure (83.7% vs. 90.1%) (Table 2).

**Table 2:** Invasive procedures in labor and birth care according to the Baby-Friendly Hospital Initiative Woman-Friendly Care criterion and their association with exclusive breastfeeding at discharge (N = 756), University Hospital, South Region of Brazil, 2020-2021

	Total		Exclusive breastfeeding at discharge		
	**n**	**% (95% CI)**	**n**	**% (95% CI)**	*p* value^^*^^
**Labor induction (use of a catheter and/or vaginal tablet and/or intravenous oxytocin)**					
No	464	61.4 (57.8-54.8)	397	85.6 (82.4-88.8)	0.622
Yes	292	38.6 (35.2-42.2)	246	84.2 (80.1-88.4)	
**Labor augmentation (intravenous oxytocin)**					
No	516	68.2 (64.8-71.4)	440	85.3 (82.2-88.3)	0.805
Yes	240	31.8 (28.5-35.2)	203	84.6 (80.0-89.2)	
**Artificial rupture of membranes**					
No	590	78.0 (74.9-80.9)	504	85.4 (82.6-88.3)	0.590
Yes	166	22.0 (19.1-25.1)	139	83.7 (78.1-89.3)	
**Perineal incision (episiotomy)†**					
No	453	93.6 (91.0-95.5)	396	87.4 (84.4-90.5)	0.959
Yes	31	6.4 (4.5-9.0)	27	87.1 (75.3-98.9)	
**Cesarean birth**					
No	484	64.0 (60.5-67.4)	423	87.4 (84.4-90.4)	**0.016**
Yes	272	36.0 (32.6-39.5)	220	80.9 (76.2-85.6)	
**Any invasive procedure listed above**					
No	161	21.3 (18.5-24.4)	145	90.1 (85.4-94.7)	**0.044**
Yes	595	78.7 (75.6-81.5)	498	83.7 (80.7-86.7)	
**Explanation of the reason for or need for the interventions‡**					
No	34	6.2 (4.5-8.6)	29	85.3 (73.4-97.2)	0.791
Yes	511	93.8 (91.4-95.5)	427	83.6 (80.3-86.8)	

* *Chi-square test; †Women who underwent cesarean birth were excluded (n = 483); ‡Women who did not undergo invasive procedures and those who did not receive information were excluded (n = 545).*

All breastfeeding-support practices implemented in the labor and delivery unit were associated with a higher prevalence of exclusive breastfeeding at discharge, including skin-to-skin contact (87.1% vs. 74.8%), recognition of feeding cues (88.6% vs. 81.4%), assistance with positioning the baby to breastfeed (88.4% vs. 72.8%), and receipt of any guidance or good practice listed above (87.6% vs. 71.7%) (Table 3).

**Table 3:** Breastfeeding-support practices implemented in the labor and delivery unit under the fourth step of the Baby-Friendly Hospital Initiative and their association with exclusive breastfeeding at discharge (N = 756), University Hospital, South Region of Brazil, 2020-2021

		Total	Exclusive breastfeeding at discharge		
	**n**	**% (95% CI)**	**n**	**% (95% CI)**	***p* value** ^^*^^
**Skin-to-skin contact**					
No	27	16.8 (14.3-19.6)	95	74.8 (67.3-82.4)	**< 0.001**
Yes	629	83.2 (80.4-85.7)	548	87.1 (84.5-89.7)	
**Recognition of feeding cues**					
No	371	49.1 (45.5-52.6)	302	81.4 (77.4-85.4)	**0.006**
Yes	385	50.1 (47.4-54.5)	341	88.6 (85.4-91.7)	
**Assistance with positioning the baby to breastfeed**					
No	162	21.4 (18.6-24.5)	118	72.8 (66.0-80.0)	**< 0.001**
Yes	594	78.6 (75.5-81.4)	525	88.4 (85.8-91.0)	
**Any guidance or good practice listed above**					
No	120	15.9 (13.4-18.7)	86	71.7 (63.6-85.0)	**< 0.001**
Yes	636	84.1 (81.3-86.6)	557	87.6 (85.0-90.1)	

* *Chi-square test.*

When breastfeeding-support practices were analyzed by mode of birth, skin-to-skin contact (p < 0.001) and guidance on recognizing feeding cues (p = 0.008) showed statistically significant differences between vaginal birth and cesarean birth. Among women who had a vaginal birth, the prevalence of skin-to-skin contact was 93.4%, compared with 65.2% among those who underwent cesarean birth. Regarding guidance on recognizing feeding cues, 54.5% of women who had a vaginal birth received this guidance, compared with 44.7% of those who underwent cesarean birth. Vaginal birth was also associated with a higher prevalence of exclusive breastfeeding at discharge (p = 0.016) (data not shown in the tables).

## DISCUSSION

In the immediate postpartum period, studies have shown that adequate support, including breastfeeding guidance and ongoing support, contributes significantly to the promotion and maintenance of exclusive breastfeeding^(5,6)^. In addition, a multidisciplinary approach involving healthcare professionals and lactation support services strengthens mothers’ confidence in their breastfeeding abilities, thereby reducing rates of early weaning^(7,8)^.

This study found a high prevalence of exclusive breastfeeding at discharge, close to that observed in a study conducted in several BFHI maternity hospitals in Italy (≥ 80.0%) under comparable conditions in terms of care setting and data collection during the COVID-19 pandemic^(5)^. Another study involving more than 26,000 mother-infant dyads from 17 European countries found a prevalence of exclusive breastfeeding at hospital discharge of 72.4% during the pandemic^(9)^. In Brazil, the prevalence of exclusive breastfeeding at discharge observed in a public maternity hospital in the state of Rio de Janeiro, in a study likewise based on data collected during the COVID-19 pandemic, was very close to that found in the present study (83.7%)^(6)^.

However, studies have shown that COVID-19 affected breastfeeding maintenance within healthcare institutions. In one hospital in Italy, the rate of exclusive breastfeeding at hospital discharge was 86.4% before the pandemic and fell to 70.4% during the pandemic^(8)^. Another study conducted in Spain compared uninfected women with women diagnosed with COVID-19 on admission for childbirth and found rates of 81.2% and 62.7% for exclusive breastfeeding at discharge, respectively^(7)^.

With regard to labor and birth care practices, it is important to understand the study context. Since its opening in the 1990s, the maternity unit under study has adopted a philosophy of care grounded in the principles of humanization and interdisciplinarity. From that point on, it began promoting changes in the prevailing obstetric model, moving away from interventionist and unnecessary practices that were routine at the time, such as enemas and pubic shaving on admission for childbirth, while encouraging good practices such as upright positions during the second stage of labor. Another practice that was innovative at the time was encouraging the presence of a companion^(10)^, given the importance of that person’s physical and emotional support throughout labor, birth, and the maternity stay^(11)^. This practice contributed to the enactment of the Companion Law, which came into force nationwide in 2005^(12)^.

However, in Brazil and around the world, there was a setback in ensuring the rights of women and their babies, especially at the beginning of the pandemic, when companions were temporarily barred, or their presence was restricted^(12-14)^. At the study institution, women were allowed a companion in the labor and delivery unit. However, to reduce circulation, postpartum women and their newborns were transferred to the rooming-in unit without a companion after birth and remained there until hospital discharge.

Despite this limitation, participants in this study reported having a companion of their choice in the labor and delivery unit at a higher rate (91.7%) than in other Brazilian studies. A study conducted in Brazilian maternity hospitals affiliated with the Stork Network before the COVID-19 pandemic showed that the presence of a companion only during childbirth was guaranteed in only 40.9% of the maternity hospitals^(15)^.

In addition to the presence of a companion, offering fluids and light foods during labor is a practice recommended by the WHO^(3)^ and reinforced in Brazilian national guidelines^(2)^. In our study, access to fluids and light foods (71.3%) was nearly three times the rate reported by Leal et al.^(16)^ (25.6%) and more than four times the rate found in another study conducted in Santa Catarina^(17)^ (16.7%). Among women who had vaginal births, our result was close to the rate reported in a study conducted at a public maternity hospital in Roraima (67.4%)^(18)^.

Another relevant aspect of good labor and birth care practices is the use of nonpharmacological pain relief methods, which help support women in labor and relieve pain. Among the benefits of these methods, an integrative review found relaxation, reduced anxiety, local analgesia in painful areas, and improved labor progression^(19)^. The prevalence of this practice varies widely across Brazilian studies, depending on the care provided. In some publications, including data from the Birth in Brazil study, the use of nonpharmacological methods ranged from 20% to 30%^(16,20,21)^. Among women who had vaginal births, the monthly rate ranged from 59.5% to 71.2% in a public maternity hospital in Roraima^(18)^.

Still within the scope of good labor and birth care practices, the Woman-Friendly Care criterion calls for an appropriate care environment, assessed in this study through a question about a calm, welcoming environment with privacy and soft lighting, to which 73.6% of the women responded positively. This was the only variable among the good labor and birth care practices that was statistically associated with exclusive breastfeeding at discharge. This may be a novel finding, given the scarcity of data from previous studies for comparison. Although this is a subjective variable that depends on each participant’s perception, one possible explanation for this association is that a safe and welcoming environment may facilitate the hormonal cascade involved in labor and birth, thereby contributing to the initiation and maintenance of breastfeeding. In addition, it is worth noting that longer or higher-risk labor, requiring closer monitoring or the presence of more healthcare professionals, may be perceived as less welcoming or as offering less privacy, which in turn may hinder breastfeeding.

In contrast to good labor and birth care practices, invasive procedures, when used without a clinical indication, compromise women’s and families’ experience and satisfaction and increase the risks and complications to which women and newborns are exposed, both in the short and long term^(7,22)^. Among the interventionist practices evaluated in this study, cesarean birth and undergoing at least one intervention-such as labor induction (mechanical or pharmacological), oxytocin use, artificial rupture of membranes, or perineal incision-were associated with a lower prevalence of exclusive breastfeeding at discharge.

Among the medications most commonly used in labor and birth care, oxytocin use has been the most prevalent^(22)^. Frequently used for labor induction or to increase the intensity and frequency of uterine contractions, its prevalence in the Birth in Brazil study was 36.4%, with the South and Southeast Regions showing the highest rates at 46.1% and 47.2%, respectively^(16)^. In this study, the prevalence of oxytocin use was 31.8% when used only for labor augmentation. This percentage was higher when women who underwent labor induction (38.6%) were also included, with labor induction defined as the use of medications such as oxytocin or misoprostol or mechanical cervical stimulation. In addition, neuroscience research has shown associations between oxytocin use during labor and effects on human behavior that may compromise breastfeeding success^(22)^.

Another practice used for labor induction or augmentation is artificial rupture of membranes, which promotes prostaglandin release. Although the WHO does not recommend this practice because of the lack of evidence of benefit and its potential risks^(3)^, this study found a prevalence of 22.0%, compared with 39.1% in national data among women in labor^(16)^. In another study conducted at a university hospital in the South Region of Brazil, the rates were 28.2% for oxytocin use and 35.9% for artificial rupture of membranes^(17)^.

Among other interventionist practices, episiotomy stands out. Despite the lack of evidence supporting its benefits, studies comparing routine and selective use have shown that, among women who did not undergo instrumental vaginal birth (forceps or vacuum extraction), selective use is associated with less severe perineal trauma^(21)^. In addition, although no protocols establish an acceptable number of episiotomies, safe childbirth care can be provided without this procedure^(22)^.

Of the participants in this study, 31 women (6.4%) reported having undergone an episiotomy. However, a study conducted at the same hospital using medical record data found a prevalence of 2.5%^(21,22)^. The difference between these values suggests the possibility of bias in both studies. In the present study, the nonprobability sample should be taken into account, as selection bias may have occurred because more women who underwent the procedure may have participated. In addition, information bias may have occurred because participants may have had difficulty distinguishing spontaneous laceration from episiotomy, which may have led them to equate the two. In the medical record-based study, instrument bias is also possible because the procedure may not have been documented. Even so, the prevalence in this study was lower than that reported in other Brazilian studies. National data identified an episiotomy rate of 53.3% among women who had a vaginal birth^(16)^, although this percentage was also higher than rates reported in studies with lower prevalences, such as in Alagoas, where it was 12.2% in 2015^(23)^, and in Roraima, where it was 8.8% between 2019 and 2020^(15)^.

With regard to the invasive procedures evaluated, 21.2% of participants reported that they had not undergone any of the interventions investigated (labor induction or augmentation, rupture of membranes, perineal incision, and cesarean birth), which may be understood as vaginal births without intervention. At the same hospital, 17.7% of births occurred without complications or interventions, according to medical record review and interviews^(24)^. In the Birth in Brazil study, the prevalence of vaginal birth without any intervention during labor and birth was only 5.6%^(20)^.

Cesarean birth was among the most prevalent obstetric interventions, with a rate of 36.1% among participants. This rate is lower than that found in the state in 2020 and 2021 (58%), even when only procedures performed within the SUS during the same study period were considered (47%)^(25,26)^. Not undergoing cesarean birth was also associated with a higher rate of exclusive breastfeeding at discharge, whereas cesarean birth was associated with a lower prevalence of skin-to-skin contact and guidance on recognizing feeding cues.

It is worth noting that breastfeeding in the first hour of life is associated with continued exclusive breastfeeding and is even considered a protective factor against neonatal mortality^(27-29)^. Colonization of the infant gut by saprophytic bacteria present in breast milk, together with the immunologic factors in maternal colostrum, has a proven protective effect on newborns^(26)^.

Among the beneficial practices in this initial period is skin-to-skin contact. It promotes regulation of the newborn’s body temperature, reduces stress, and increases the likelihood of exclusive breastfeeding, contributing to a healthier start in life. In this study, skin-to-skin contact was reported by 83.2% of participants, a percentage similar to that reported in a multinational study involving 17 European countries conducted during the pandemic (88.7%)^(9)^.

When comparing the prevalence of skin-to-skin contact between women who had a vaginal birth (93.4%) and those who underwent cesarean birth (65.2%), it is noteworthy that, although lower in the cesarean group, the prevalence was still higher than the national rate reported in Stork Network hospitals, where 43.2% of newborns had skin-to-skin contact after cesarean birth^(27)^. These results also exceed those of a study conducted in Maranhão at a Baby-Friendly Hospital, in which only 27.3% of mother-infant dyads had immediate skin-to-skin contact after cesarean birth^(30)^. These advances reflect greater awareness of the benefits of this practice, especially after cesarean birth. However, adherence still needs to increase, and its broad implementation must be ensured across all modes of birth and in all institutions.

In the present study, skin-to-skin contact stood out as a key practice. In addition, encouraging early breastfeeding and providing appropriate guidance are known to be essential for successful exclusive breastfeeding, not only at hospital discharge but throughout the first 6 months of life, as well as for continued breastfeeding until 2 years of age or older^(29)^. When breastfeeding-support practices are considered alongside the challenges healthcare professionals faced during the COVID 19 pandemic, the need to improve care becomes evident. It is important to expand guidance in the delivery room, such as how to identify signs that the baby is ready to feed, and to strengthen all practices that support breastfeeding, especially for women who undergo cesarean birth. Continuing education and the development of protocols to standardize care may help improve care, since healthcare professionals may understand and implement these practices differently^(30)^.

In this context, the leading role of obstetric nurses in breastfeeding promotion, protection, and support should be emphasized, particularly given their direct and continuous involvement in caring for women and newborns during labor and birth. Brazilian and international evidence indicates that models of childbirth care led or strongly supported by obstetric nurses are associated with greater use of good practices, such as offering food, encouraging movement, and using nonpharmacological pain relief methods during labor, as well as with a higher likelihood of spontaneous vaginal birth and lower use of interventions such as the lithotomy position, fundal pressure, and episiotomy at birth^(31,32)^. For these reasons, these professionals can play a strategic role both in identifying barriers to breastfeeding initiation-particularly in more vulnerable situations, such as cesarean birth-and in providing high-quality guidance, emotional support, and encouragement of good practices starting in the delivery room.

### Study limitations

The study was conducted at an institution recognized for the quality of its care and included a substantial sample of 756 women. However, participants self-selected into the study, resulting in a nonprobability sample that limits the generalizability of the findings and requires cautious interpretation because it is subject to biases common to observational studies. Information and recall bias are possible, since the data were self-reported and reflected participants’ perceptions of the care practices they received. In addition, data were collected using an electronic form, which may have excluded individuals with limited internet access. It should also be noted that no sociodemographic data or data on clinical and obstetric complications were collected, which made it impossible to perform association analyses adjusted for potential confounders.

### Contributions to the field

This study reinforces the evidence on the benefits of the Woman-Friendly Care criterion for strengthening breastfeeding. It highlights it as an essential guide for improving care and reorienting the historically dominant, provider-centered obstetric model toward woman-centered, evidence-based care. The findings show that adopting care practices that respect the physiology of labor and limit unnecessary interventions favors positive outcomes for women, newborns, and the healthcare team. These practices are closely linked to the role of obstetric nurses. By highlighting the importance of BFHI practices in breastfeeding promotion, protection, and support, the study also underscores the strategic potential of nursing to lead care quality assessment and improvement initiatives in hospitals and maternity units, thereby contributing to greater adherence to and implementation of the steps recommended by the BFHI and by public policies for obstetric and neonatal care.

## CONCLUSIONS

Labor and birth care practices, such as ensuring privacy and a welcoming environment, were directly associated with breastfeeding success. The same was true of breastfeeding-support practices at birth, including skin-to-skin contact, recognition of feeding cues, and assistance with positioning the baby to breastfeed in the labor and delivery unit. These findings reinforce the importance of public policies such as the BFHI and the Woman-Friendly Care criterion, which ensure more respectful, woman-centered, evidence-based care. For these principles to become established in practice, healthcare professionals should remain committed to advancing this model of care. This contributes to improving care for women and newborns, improving maternal and child health indicators, and supporting healthy child development from the first days of life.

## Data Availability

The research data are available in a repository: https://doi.org/10.48331/scielodata.OALXWW.
