# Supplementary material for: Labor and birth care practices associated with exclusive breastfeeding at discharge
Source: Rev Bras Enferm. 2026 Sep 21;79:e20250305. doi: 10.1590/0034-7167-2025-0305 (PMC13592783; doi:10.1590/0034-7167-2025-0305)
Supplement: Translation

# Translation: Práticas de assistência ao parto e nascimento relacionadas com a alta em aleitamento materno exclusivo

**DOI:** 10.1590/0034-7167-2025-0305pt

**Authors:** Manuela Beatriz Velho, Isabel Cristina Alves Maliska, Zannis Benevides de Andrade, Franciane Zabloski Vieira, Luana Costa Albino, Thayná Ventura, Liliana Maria Ferreira Vicente, Roxana Knobel

**Affiliations:** I Universidade Federal de Santa Catarina. Florianópolis, Santa Catarina, Brasil

**Keywords:** Trabalho de Parto, Assistência Obstétrica, Prática Profissional, Aleitamento Materno, Política de Saúde.

## Abstract

**Objetivos:**

identificar a prevalência de práticas de assistência ao parto e nascimento e sua relação com a alta em aleitamento materno exclusivo.

**Métodos:**

estudo transversal baseado no critério global Cuidado Amigo da Mulher, com 756 puérperas de um Hospital Amigo da Criança. Calcularam-se as proporções e os ICs 95% e aplicou-se teste qui-quadrado.

**Resultados:**

a prevalência de alta em aleitamento materno exclusivo foi de 85,0%. Também tiveram maior prevalência e associação com o desfecho: acesso a boas práticas como privacidade e ambiente acolhedor (73,9%), contato pele a pele (83,2%) e auxílio na colocação do bebê para mamar (78,6%). Entre os procedimentos invasivos, a cesariana (36,0%) foi associada a menor prevalência do desfecho.

**Conclusões:**

práticas de assistência ao parto foram associadas à alta em aleitamento materno exclusivo, ressaltando a importância de políticas públicas que garantam atendimento humanizado e baseado em evidências.

## INTRODUÇÃO

A Iniciativa Hospital Amigo da Criança (IHAC) integra ações voltadas ao critério global Cuidado Amigo da Mulher, incorporado como critério dessa política no Brasil em 2014. Denominado em outros países como “Cuidado Amigo da Mãe”, prevê uma série de cuidados à mulher durante o trabalho de parto e nascimento a fim favorecer a evolução do trabalho de parto de forma natural. Entre esses cuidados, destacam-se: garantia de um acompanhante de sua livre escolha; oferta de líquidos e alimentos leves durante o trabalho de parto; incentivo à livre movimentação durante o trabalho de parto, bem como adotar sua melhor posição para o parto; ambiente adequado, tranquilo e acolhedor, com privacidade e iluminação suave; acesso a métodos não farmacológicos de alívio da dor; oferta de cuidados que reduzem procedimentos invasivos, a menos que necessários em razão de complicações, sendo tal fato devidamente explicado à mulher; e, por fim, autorização da presença de doula em apoio à mulher de forma contínua, se for da sua vontade^(1)^.

Para qualificar o atendimento às mulheres e suas famílias no momento do nascimento dos filhos, o Ministério da Saúde do Brasil publicou, em 2016, as Diretrizes Nacionais de Assistência ao Parto Normal^(2)^; já a Organização Mundial da Saúde (OMS), em 2018, publicou as recomendações de cuidado intraparto para uma experiência positiva^(3)^. Essas diretrizes apresentam informações científicas que orientam as equipes em relação às práticas de assistência ao parto e ao nascimento. Seu propósito é diminuir o uso de intervenções desnecessárias, promover a adoção de boas práticas e contribuir com a redução das elevadas taxas de operação cesariana, que constituem uma preocupação na realidade brasileira^(2)^.

Assim, as práticas de assistência ao parto destacam-se na promoção, proteção e apoio ao aleitamento materno. Além disso, uma assistência qualificada gera melhores condições de saúde tanto para as mulheres quanto para as crianças, alinhando-se ao terceiro Objetivo do Desenvolvimento Sustentável, que visa garantir uma vida saudável e promover o bem-estar para todos, em todas as idades^(4)^.

Ao compreender a necessidade de assegurar o acesso das mulheres a essas práticas e de promover o aleitamento materno, elaborou-se a seguinte questão de pesquisa: Qual é a prevalência das práticas de assistência ao parto e nascimento propostas no critério global Cuidado Amigo da Mulher e se existe relação dessas práticas com a alta em aleitamento materno exclusivo?

## OBJETIVOS

Identificar a prevalência das práticas de assistência ao parto e nascimento e sua relação com a alta em aleitamento materno exclusivo.

## MÉTODOS

### Aspectos éticos

O estudo foi conduzido conforme diretrizes éticas nacionais e internacionais e foi aprovado por um Comitê de Ética em Pesquisa com Seres Humanos sob o CAAE número 36214720.0.0000.0121, com a devida autorização da instituição participante. A pesquisa não recebeu financiamento externo.

### Desenho, período e local do estudo

Trata-se de um estudo quantitativo, transversal, realizado em ambiente virtual. A elaboração deste relatório de pesquisa foi guiado pelas diretrizes do *Strengthening the reporting of observational studies in epidemiology* (STROBE). O estudo foi conduzido na maternidade de um hospital-escola localizado na Região Sul do Brasil, entre julho de 2020 e setembro de 2021. A maternidade investigada possui, desde 1997, o título de Hospital Amigo da Criança e adota uma filosofia de atendimento fundamentada nos princípios de humanização e interdisciplinaridade. Disponibiliza aos usuários atendimentos na emergência obstétrica, centro obstétrico, unidade ginecológica, alojamento conjunto e unidade de terapia intensiva neonatal, todos com atendimento exclusivo pelo Sistema Único de Saúde (SUS). A assistência é prestada por uma equipe multidisciplinar em todos os seus espaços. A instituição oferece o programa de residência médica em obstetrícia, ginecologia e pediatria, além de residência integrada multiprofissional em saúde, com ênfase em saúde da mulher e da criança.

Nas unidades da maternidade, o centro obstétrico disponibiliza cinco leitos de pré-parto, sendo três adaptados como Pré-Parto/Parto/Puerpério (PPP), bem como um leito de isolamento, duas salas cirúrgicas e dois leitos de recuperação pós-anestésica. O alojamento conjunto possui 17 leitos de internação para o atendimento de puérperas. A maternidade conta com uma equipe de enfermagem responsável pela Central de Incentivo ao Aleitamento Materno (CIAM), que acompanha e reforça as orientações relacionadas ao aleitamento materno.

### População, critérios de inclusão e exclusão

A população do estudo foram todas as mulheres que tiveram o nascimento de seus filhos na maternidade investigada durante o período do estudo. Os critérios de inclusão foram ter idade igual ou superior a 18 anos e internação do binômio mãe-bebê em alojamento conjunto, após o nascimento. Foram excluídas as mulheres que não informaram um número de telefone para contato na ocasião do atendimento pela equipe do CIAM, durante a internação no Alojamento Conjunto.

Entre 24 e 48 horas após a alta da maternidade, as mulheres foram contatadas por aplicativo de mensagens para receber suporte remoto em amamentação, oferecido pela equipe da CIAM durante 15 a 20 dias. Ao final desse período, o convite para participar da pesquisa foi realizado por meio do mesmo aplicativo. A coleta de dados ocorreu com o preenchimento de um questionário on-line, encaminhado pelo aplicativo de mensagem. Vale lembrar que a pesquisa foi desenvolvida durante a pandemia de covid-19.

### Variáveis

A variável desfecho investigada foi a alta em aleitamento materno exclusivo, enquanto as variáveis de exposição foram as práticas de assistência ao parto e nascimento, categorizadas em boas práticas (possibilidade de ter um acompanhante de sua livre escolha no centro obstétrico, acesso a líquidos e alimentos leves, uso de bola suíça, utilização de cavalinho, estímulo para realização de massagens, banho prolongado no chuveiro, privacidade e ambiente acolhedor, orientação e incentivo ao acompanhante para participação nos cuidados e escolha da melhor posição para o parto) e o agrupamento das boas práticas na variável “alguma orientação ou boa prática citada anteriormente”; procedimentos invasivos (indução do trabalho de parto [uso de sonda e/ou comprimido via vaginal e/ou uso de soro com ocitocina], aceleração do trabalho de parto [uso de soro com ocitocina], rompimento da bolsa intencional realizado pelo profissional, corte no períneo [episiotomia], operação cesariana) e o agrupamento dos procedimentos invasivos na variável “algum procedimento invasivo citado anteriormente”, além da variável “explicação sobre o motivo ou necessidade das intervenções”; e práticas que favorecem o aleitamento materno (contato pele a pele imediatamente após o nascimento ou nos primeiros cinco minutos de vida com duração de pelo menos uma hora, identificação dos sinais de que o bebê quer mamar e auxílio na colocação do bebê para mamar) realizadas no centro obstétrico.

### Análise de dados

Os dados coletados foram organizados numa planilha eletrônica e analisados no software estatístico Stata (versão 13). Todas as variáveis foram categorizadas de forma qualitativa nominal (sim ou não). Na análise de dados, foram apresentadas as frequências absolutas, calculados as proporções e os intervalos de confiança de 95% (IC 95%) das práticas, seguidos das frequências absolutas, proporções e ICs 95% das práticas e sua relação com a alta em aleitamento materno. Nas práticas que favorecem o aleitamento materno, foram investigadas associações com a via de nascimento (parto normal e operação cesariana). O teste de qui-quadrado foi aplicado para identificar uma possível diferença estatística entre as proporções segundo os desfechos, considerando significativos os valores de *p* < 0,05.

## RESULTADOS

No período do estudo, foram registrados 3.080 nascimentos na instituição. As 2.455 mulheres que atenderam aos critérios de inclusão foram contatadas por aplicativo de mensagens e receberam o questionário, correspondendo a 79,7% das mulheres atendidas na maternidade. Daquelas que receberam o questionário, a pesquisa teve o retorno de 756, o que representa a uma taxa de resposta de 30,8% entre as mulheres elegíveis e a 24,5% do total de mulheres atendidas no mesmo período (Figura 1).

**Figura 1:** Fluxograma de seleção das participantes do estudo sobre práticas de assistência ao parto e nascimento conforme o critério global Cuidado Amigo da Mulher da Iniciativa Hospital Amigo da Criança (n = 756), Hospital Universitário, Região Sul do Brasil, 2020-2021

Entre as participantes, 643 tiveram alta em aleitamento materno exclusivo, representando uma prevalência de 85,0%. Foi encontrada uma elevada prevalência de boas práticas, sendo que a grande maioria das participantes teve acesso a pelo menos uma das boas práticas ou orientações sobre as boas práticas (97,7%). Na investigação de associações com as boas práticas, ter tido privacidade e um ambiente acolhedor apresentou maior prevalência de alta em aleitamento materno exclusivo (87,3% versus 78,7%) (Tabela 1).

**Tabela 1:** Boas práticas de assistência ao parto e nascimento conforme o critério global Cuidado Amigo da Mulher da Iniciativa Hospital Amigo da Criança e sua relação com a alta em aleitamento materno exclusivo (N = 756), Hospital Universitário, Região Sul do Brasil, 2020-2021

	Total		Alta em aleitamento materno exclusivo		
	n	% (IC 95%)	n	% (IC 95%)	Valor de *p* ^^*^^
**Acompanhante de sua livre escolha**					
Não	63	8,3 (6,6-10,5)	51	81,0 (71,3-90,6)	0,340
Sim	693	91,7 (89,5-93,4)	592	85,4 (82,8-88,1)	
**Acesso a líquidos e alimentos leves**					
Não	217	28,7 (25,6-32,0)	180	83,0 (77,9-88,0)	0,303
Sim	539	71,3 (68,0-74,4)	463	85,9 (83,0-88,8)	
**Uso de bola suíça (bola de pilates)**					
Não	244	32,3 (29,0-35,7)	207	84,8 (80,3-89,3)	0,908
Sim	512	67,7 (64,3-71,0)	436	85,2 (82,1-88,2)	
**Utilização de cavalinho**					
Não	639	84,5 (81,8-86,9)	543	85,0 (82,2-87,7)	0,891
Sim	117	15,5 (13,1-18,2)	100	85,5 (79,1-91,9)	
**Estímulo para realização de massagens**					
Não	467	61,8 (58,2-65,2)	393	84,1 (80,8-87,5)	0,378
Sim	289	38,2 (34,8-41,2)	250	86,5 (82,6-90,4)	
**Banho prolongado no chuveiro**					
Não	243	32,1 (28,9-35,6)	204	84,0 (79,3-88,6)	0,559
Sim	513	67,9 (64,4-71,1)	439	85,6 (82,5-88,6)	
**Privacidade e ambiente acolhedor**					
Não	197	26,1 (23,0-29,3)	155	78,7 (73,0-84,4)	**0,004**
Sim	559	73,9 (70,7-77,0)	488	87,3 (84,5-90,1)	
**Orientação e incentivo ao acompanhante para participação nos cuidados**					
Não	312	41,3 (37,8-44,8)	259	83,0 (78,8-87,2)	0,187
Sim	444	58,7 (55,2-62,2)	384	86,5 (83,3-90,0)	
**Escolha da sua melhor posição para o parto** ^†^					
Não	93	19,2 (15,9-23,0)	78	83,9 (76,4-91,3)	0,254
Sim	391	80,8 (77,0-84,1)	345	88,2 (85,1-81,4)	
**Alguma orientação ou boa prática anterior**					
Não	17	2,3 (1,4-3,6)	12	70,6 (48,9-92,2)	0,091
Sim	739	97,7 (96,4-98,6)	631	85,4 (82,8-87,9)	

* *Teste de qui-quadrado; †Foram excluídas as mulheres submetidas a cesariana (n = 484).*

Quanto aos procedimentos invasivos, três em cada quatro participantes relataram que foram submetidas a pelo menos um procedimento invasivo (78,7%), mas disseram que receberam explicações sobre o motivo ou a necessidade das intervenções (93,8%). Foi identificada associação com uma menor prevalência de alta em aleitamento materno exclusivo nas mulheres submetidas à operação cesariana (80,9% versus 87,4%) ou submetidas a pelo menos um procedimento invasivo (83,7% versus 90,1%) (Tabela 2).

**Tabela 2:** Procedimentos invasivos realizados na atenção ao parto e nascimento conforme o critério global Cuidado Amigo da Mulher da Iniciativa Hospital Amigo da Criança e sua relação com a alta em aleitamento materno exclusivo (N = 756), Hospital Universitário, Região Sul do Brasil, 2020-2021

	Total		Alta em aleitamento materno exclusivo		
	**n**	**% (IC 95%)**	**n**	**% (IC 95%)**	Valor de *p* ^^*^^
**Indução do trabalho de parto (uso de sonda e/ou comprimido via vaginal e/ou uso de soro com ocitocina)**					
Não	464	61,4 (57,8-54,8)	397	85,6 (82,4-88,8)	0,622
Sim	292	38,6 (35,2-42,2)	246	84,2 (80,1-88,4)	
**Aceleração do trabalho de parto (uso de soro com ocitocina)**					
Não	516	68,2 (64,8-71,4)	440	85,3 (82,2-88,3)	0,805
Sim	240	31,8 (28,5-35,2)	203	84,6 (80,0-89,2)	
**Rompimento da bolsa intencional realizado pelo profissional**					
Não	590	78,0 (74,9-80,9)	504	85,4 (82,6-88,3)	0,590
Sim	166	22,0 (19,1-25,1)	139	83,7 (78,1-89,3)	
**Corte no períneo (episiotomia)**†					
Não	453	93,6 (91,0-95,5)	396	87,4 (84,4-90,5)	0,959
Sim	31	6,4 (4,5-9,0)	27	87,1 (75,3-98,9)	
**Operação cesariana**					
Não	484	64,0 (60,5-67,4)	423	87,4 (84,4-90,4)	**0,016**
Sim	272	36,0 (32,6-39,5)	220	80,9 (76,2-85,6)	
**Algum procedimento invasivo anterior**					
Não	161	21,3 (18,5-24,4)	145	90,1 (85,4-94,7)	**0,044**
Sim	595	78,7 (75,6-81,5)	498	83,7 (80,7-86,7)	
**Explicação sobre o motivo ou necessidade das intervenções**‡					
Não	34	6,2 (4,5-8,6)	29	85,3 (73,4-97,2)	0,791
Sim	511	93,8 (91,4-95,5)	427	83,6 (80,3-86,8)	

* *Teste de qui-quadrado; †Foram excluídas as mulheres submetidas à cesariana (n = 483); ‡Foram excluídas as mulheres que não passaram por procedimentos invasivos e aquelas que não receberam informação (n = 545).*

Todas as práticas que favorecem o aleitamento materno realizadas no centro obstétrico apresentaram associação com maior prevalência de alta em aleitamento materno exclusivo, como promover contato pele a pele (87,1% versus 74,8%), identificar os sinais de que o bebê quer mamar (88,6% versus 81,4%), auxílio na colocação do bebê para mamar (88,4% versus 72,8%) e ter recebido alguma orientação ou boa prática anterior (87,6% versus 71,7%) (Tabela 3).

**Tabela 3:** Práticas que favorecem o aleitamento materno realizadas no centro obstétrico conforme o quarto passo da Iniciativa Hospital Amigo da Criança e sua relação com a alta em aleitamento materno exclusivo (N = 756), Hospital Universitário, Região Sul do Brasil, 2020-2021

		Total	Alta em aleitamento materno exclusivo		
	**n**	**% (IC 95%)**	**n**	**% (IC 95%)**	**Valor de *p*** ^^*^^
**Contato pele a pele**					
Não	27	16,8 (14,3-19,6)	95	74,8 (67,3-82,4)	**< 0,001**
Sim	629	83,2 (80,4-85,7)	548	87,1 (84,5-89,7)	
**Identificação dos sinais de que o bebê quer mamar**					
Não	371	49,1 (45,5-52,6)	302	81,4 (77,4-85,4)	**0,006**
Sim	385	50,1 (47,4-54,5)	341	88,6 (85,4-91,7)	
**Auxílio na colocação do bebê para mamar**					
Não	162	21,4 (18,6-24,5)	118	72,8 (66,0-80,0)	**< 0,001**
Sim	594	78,6 (75,5-81,4)	525	88,4 (85,8-91,0)	
**Alguma orientação ou boa prática anterior**					
Não	120	15,9 (13,4-18,7)	86	71,7 (63,6-85,0)	**< 0,001**
Sim	636	84,1 (81,3-86,6)	557	87,6 (85,0-90,1)	

* *Teste de qui-quadrado.*

Na investigação das práticas que favorecem o aleitamento materno exclusivo segundo a via de nascimento (parto normal ou operação cesariana), apresentaram diferença estatística a realização do contato pele a pele (p < 0,001) e recebimento de orientações para identificar os sinais de que o bebê quer mamar (p = 0,008). Entre as mulheres que tiveram parto vaginal, a prevalência do contato pele a pele foi de 93,4% ante 65,2% entre aquelas submetidas à cesariana. Quanto às orientações para identificar os sinais de que o bebê quer mamar, 54,5% das mulheres que tiveram um parto vaginal receberam essa orientação, em comparação com 44,7% daquelas submetidas à cesariana. Também foi identificada associação entre não realização de cesariana e maior taxa de alta em aleitamento materno exclusivo (p = 0,016) (dados não apresentados nas tabelas).

## DISCUSSÃO

No período pós-parto imediato, estudos têm demonstrado que um suporte adequado, incluindo orientação e acompanhamento contínuo sobre amamentação, contribuem significativamente para a promoção e manutenção do aleitamento materno exclusivo^(5,6)^. Além disso, uma abordagem multidisciplinar, que envolva profissionais de saúde e serviços de apoio à lactação, fortalece a confiança da mãe em suas habilidades de amamentação, reduzindo assim as taxas de desmame precoce^(7,8)^.

Este estudo encontrou uma elevada prevalência de alta em aleitamento materno exclusivo, próxima à observada em um estudo realizado em diversas maternidades IHAC na Itália (≥ 80,0%), conduzido em condições comparáveis quanto ao cenário assistencial e ao período de coleta, durante a pandemia de covid-19^(5)^. Outro estudo que envolveu mais de 26 mil binômios de 17 países europeus encontrou uma prevalência de aleitamento exclusivo na alta hospitalar de 72,4% em meio à pandemia^(9)^. Na realidade brasileira, a prevalência de alta em aleitamento materno exclusivo observada em uma maternidade pública no estado do Rio de Janeiro, em estudo também realizado com dados coletados durante a pandemia de covid-19, foi muito próxima à encontrada neste estudo (83,7%)^(6)^.

Entretanto, pesquisas mostraram o impacto da covid-19 na manutenção do aleitamento materno dentro das instituições de saúde. Num hospital da Itália, a taxa de aleitamento exclusivo na alta hospitalar era de 86,4% antes da pandemia e reduziu para 70,4% na pandemia^(8)^. Outro trabalho conduzido na Espanha comparou mulheres não infectadas e mulheres diagnosticadas com covid-19 na internação por motivo de nascimento e identificou percentuais de 81,2% e 62,7% na alta em aleitamento materno exclusivo, respectivamente^(7)^.

No tocante às práticas de assistência ao parto e nascimento, é importante compreender o contexto do estudo. Desde sua inauguração na década de 1990, maternidade investigada adota uma filosofia de assistência com base nos princípios de humanização e interdisciplinaridade. Dali em diante, passou a promover mudanças no modelo obstétrico hegemônico, deixando de adotar práticas intervencionistas e desnecessárias que, à época, eram rotineiras, como a lavagem intestinal e a tricotomia na admissão das mulheres para o parto, e incentivando boas práticas, como o estímulo a posições verticalizadas no período expulsivo. Outra prática que, à época, era inovadora foi o estímulo à presença de um acompanhante^(10)^, em razão da importância de seu apoio físico e emocional durante todo o processo de nascimento e a permanência na maternidade^(11)^. Essa prática contribuiu para a criação da lei que passou a vigorar nacionalmente em 2005, conhecida como Lei do Acompanhante^(12)^.

No entanto, no Brasil e no mundo, observou-se um retrocesso na garantia dos direitos das mulheres e seus bebês, principalmente no início da pandemia, quando ocorreram períodos de proibição da presença de acompanhante ou da limitação de sua presença^(12-14)^. Na instituição pesquisada, a presença do acompanhante foi permitida no centro obstétrico; contudo, para reduzir a circulação de pessoas, optou-se por encaminhar as puérperas e recém-nascidos para o alojamento conjunto sem acompanhante após o nascimento, permanecendo assim até a alta hospitalar.

Mesmo com essa limitação, as participantes deste estudo relataram que tiveram a presença do acompanhante de sua escolha no centro obstétrico, com percentuais mais elevados (91,7%) do que em outras pesquisas nacionais. Estudo realizado em maternidades brasileiras vinculadas à Rede Cegonha, antes da pandemia de covid-19, mostrou que a presença de acompanhante somente no parto foi garantida em apenas 40,9% das maternidades^(15)^.

Além da presença de acompanhante, a oferta de líquidos e alimentos leves durante o trabalho de parto também constitui uma prática recomendada pela OMS^(3)^ e reforçada nas diretrizes nacionais brasileiras^(2)^. Em nosso estudo, o acesso a líquidos e alimentos leves (71,3%) foi quase três vezes maior (25,6%) que o relatado no estudo de Leal e colaboradores^(16)^ e quatro vezes maior (16,7%) que o percentual encontrado em pesquisa também realizada em Santa Catarina^(17)^. Na comparação com mulheres que tiveram apenas partos vaginais, nossos resultados foram próximos do percentual descrito em estudo conduzido numa maternidade pública localizada em Roraima (67,4%)^(18)^.

Outro aspecto relevante dentro das boas práticas de assistência ao parto consiste na utilização de métodos não farmacológicos para alívio da dor, o que contribui para dar suporte e controlar a sensação de dor nas parturientes. Entre os benefícios destes métodos, um estudo de revisão integrativa encontrou o relaxamento, a diminuição dos níveis de ansiedade, a analgesia local de regiões afetadas pela dor e uma melhor progressão do trabalho de parto^(19)^. Esse tipo de cuidado varia amplamente em estudos brasileiros e conforme assistência prestada. Em algumas publicações, incluindo dados da pesquisa Nascer no Brasil, uso de métodos não farmacológicos variou de 20% a 30%^(16,20,21)^. Já entre mulheres com parto vaginal, esse percentual mensal variou entre 59,5% e 71,2% em maternidade pública localizada em Roraima^(18)^.

Ainda no campo das boas práticas de assistência ao parto, o critério global Cuidado Amigo da Mulher prevê a oferta de uma ambiência adequada, avaliada neste estudo mediante a pergunta sobre um ambiente tranquilo e acolhedor, com privacidade e iluminação suave, à qual 73,6% das entrevistadas responderam positivamente. Esta foi a única variável dentre as boas práticas de assistência ao parto que apresentou significância estatística com a alta em aleitamento materno exclusivo. Trata-se de um achado potencialmente inédito, haja vista a escassez de dados em estudos anteriores para comparação. Apesar de ser uma variável subjetiva que depende da percepção de cada participante, uma possível explicação para essa associação é que um ambiente seguro e acolhedor pode facilitar a cascata hormonal envolvida no processo de nascimento, contribuindo assim para o início e manutenção do aleitamento materno. Além disso, é válido pontuar que um trabalho de parto mais prolongado ou de maior risco, que exija maior monitoramento ou a presença de mais profissionais, possa ser interpretado como menos acolhedor ou com menor privacidade, o que, por sua vez, pode dificultar o aleitamento materno.

Em contraposição às boas práticas de assistência ao parto, os procedimentos invasivos, quando utilizados sem uma indicação clínica, interferem na experiência e satisfação das mulheres e suas famílias, além de aumentarem os riscos e complicações a que estão sendo expostos as mulheres e os recém-nascidos, tanto no curto quanto no longo prazo^(7,22)^. Entre as práticas intervencionistas avaliadas neste estudo, a realização de cesariana ou a submissão a pelo menos uma intervenção - como indução do trabalho de parto (mecânico ou farmacológico), uso de ocitocina e rompimento artificial das membranas ou corte no períneo - estiveram associadas a menor prevalência de alta em aleitamento materno exclusivo.

Dentre as medicações mais utilizadas na atenção ao parto e nascimento, a ocitocina tem sido a mais prevalente^(22)^. Frequentemente utilizada em casos de indução do trabalho de parto ou para aumentar a intensidade e a frequência das contrações uterinas, a prevalência identificada na pesquisa Nascer no Brasil foi de 36,4%, sendo as Regiões Sul e Sudeste as de maior prevalência, com 46,1% e 47,2%, respectivamente^(16)^. Neste estudo, foi encontrada prevalência de 31,8% no uso da ocitocina, indicada apenas para acelerar o trabalho de parto. Esse percentual foi maior quando consideradas as mulheres que foram submetidas à indução do trabalho de parto (38,6%), caracterizada pelo uso de medicações como a ocitocina ou o misoprostol, ou pelo estímulo mecânico da cérvix uterina. Ademais, pesquisas na área de neurociências têm demonstrado associações entre o uso da ocitocina no trabalho de parto e efeitos no comportamento humano, que podem comprometer o sucesso da amamentação^(22)^.

Outra prática realizada para induzir ou acelerar o trabalho de parto é o rompimento artificial de membranas, pela liberação de prostaglandinas. Apesar de ser uma prática não recomendada pela OMS pela ausência de benefícios da intervenção e seus potenciais riscos^(3)^, os resultados deste estudo mostraram uma prevalência de 22%, enquanto dados nacionais revelaram uma prevalência de 39,1% entre as mulheres em trabalho de parto^(16)^. Na comparação com outro estudo conduzido em hospital universitário da Região Sul do Brasil, os percentuais foram de 28,2% no uso de ocitocina e 35,9% no rompimento artificial das membranas^(17)^.

Entre outras práticas intervencionistas, destaca-se a episiotomia. A despeito da inexistência de evidências que comprovem seus benefícios, estudos que compararam a realização de rotina versus seletiva mostraram que, em mulheres sem o uso de parto instrumental (fórceps ou vácuo extrator), a prática seletiva está associada a menor trauma perineal grave^(21)^. Além disso, embora não existam protocolos com um número aceitável de episiotomias, é possível o atendimento ao parto seguro sem a utilização desse procedimento^(22)^.

Das participantes deste estudo, 31 (6,4%) mulheres relataram ter sido submetidas a corte no períneo (episiotomia). Entretanto, estudo realizado no mesmo hospital, com pesquisa em prontuários, encontrou uma prevalência de 2,5%^(21,22)^. A diferença entre os valores sugere a possibilidade de vieses em ambos os estudos. No presente estudo, deve-se considerar a amostra não probabilística, com potencial viés de seleção, já que podem ter participado mais mulheres submetidas ao procedimento. Além disso, pode ter ocorrido viés de informação, devido à dificuldade em diferenciar laceração espontânea de episiotomia, o que pode ter levado as participantes a equiparar ambas. No estudo baseado em prontuários, por sua vez, há a possibilidade de viés do instrumento, em razão da ausência de registro do procedimento. Ainda assim, a prevalência neste estudo foi menor que a relatada em outras pesquisas no Brasil. Dados nacionais identificaram uma taxa de episiotomia de 53,3% entre as mulheres que tiveram parto vaginal^(16)^, embora esse percentual também seja superior ao descrito em estudos com menores prevalências, como em Alagoas, com 12,2% em 2015^(23)^, e em Roraima, com 8,8% entre 2019 e 2020^(15)^.

No que se refere aos procedimentos invasivos avaliados, 21,2% das participantes informaram que não foram submetidas a nenhuma intervenção pesquisada (indução ou aceleração do trabalho de parto, rompimento da bolsa, corte no períneo e operação cesariana), o que se compreende como partos normais sem intervenção. Uma taxa de 17,7% de partos sem nenhuma complicação/intervenção foi encontrada no mesmo hospital, mas com revisão de prontuários e entrevistas^(24)^. Na pesquisa Nascer no Brasil, a prevalência do parto normal sem qualquer intervenção durante o trabalho de parto e parto foi de apenas 5,6%^(20)^.

A operação cesariana esteve entre as intervenções obstétricas mais prevalentes, com uma taxa de 36,1% entre as participantes. Essa taxa é inferior à encontrada no estado nos anos de 2020 e 2021 (58%), mesmo quando avaliados somente os procedimentos realizados no SUS, no mesmo período do estudo (47%)^(25,26)^. Observou-se também que a não realização de cesariana esteve associada a uma maior taxa de alta em aleitamento materno exclusivo, enquanto a ocorrência desse procedimento se associou a uma menor prevalência de contato pele a pele e de orientações sobre os sinais de que o bebê quer mamar.

Deve-se destacar que a amamentação na primeira hora de vida está relacionada com a continuidade do aleitamento materno exclusivo, por isso é, inclusive, considerada um fator protetor para a mortalidade neonatal^(27-29)^. A colonização intestinal de bactérias saprófitas encontradas no leite materno e os fatores imunológicos presentes no colostro materno têm efeito protetor comprovado para o recém-nascido^(26)^.

Entre as práticas benéficas nesse momento inicial, está o contato pele a pele. Ele promove a regulação da temperatura corporal do recém-nascido, reduz o estresse e aumenta as chances de amamentação exclusiva, contribuindo para um início mais saudável da vida do bebê. Neste estudo, o contato pele a pele ocorreu em 83,2% da nossa amostra - porcentagem semelhante à encontrada em estudo multinacional com participação de 17 países europeus, realizado durante a pandemia (88,7%)^(9)^.

Ao comparar a prevalência do contato pele a pele entre as mulheres que tiveram parto vaginal (93,4%) e aquelas submetidas à cesariana (65,2%), observa-se que, embora inferior na cesariana, a prevalência ainda foi superior aos dados nacionais encontrados em hospitais da Rede Cegonha, no quais 43,2% dos recém-nascidos realizaram contato pele a pele após cesariana^(27)^. Esses resultados também superam os dados do estudo conduzido no Maranhão, em um Hospital Amigo da Criança, no qual apenas 27,3% das díades mãe e filho tiveram contato pele a pele imediato após a cesariana^(30)^. Esses avanços refletem uma maior conscientização sobre os benefícios dessa prática, especialmente na cesariana, embora ainda haja necessidade de aumentar a adesão e garantir sua ampla implementação em todas as modalidades de nascimento e em todas as instituições.

No presente estudo, a prática do contato pele a pele teve destaque. Além disso, o estímulo à amamentação precoce e as orientações adequadas são sabidamente fundamentais para um desfecho positivo em relação à amamentação exclusiva, não apenas na alta hospitalar, mas até os 6 meses de vida e continuada até os 2 anos ou mais^(29)^. Por outro lado, ao considerar as práticas que favorecem o aleitamento materno, levando em consideração os desafios enfrentados também pelos profissionais de saúde na vigência da pandemia de covid-19, evidencia-se a necessidade de aprimoramento da assistência. Destaca-se a importância de maior oferta de orientações na sala de parto, como identificar os sinais de prontidão do bebê para mamar, bem como de todas as práticas que promovem o aleitamento materno, sobretudo para as mulheres submetidas à operação cesariana. A educação permanente pode ser uma estratégia para qualificar a assistência, assim como a elaboração de protocolos voltados à padronização da assistência, visto que, muitas vezes, as práticas podem ser entendidas e aplicadas de diferentes formas pelos profissionais^(30)^.

Nesse contexto, destaca-se o protagonismo da enfermagem obstétrica na promoção, proteção e apoio ao aleitamento materno, especialmente por causa da sua atuação direta e contínua no cuidado à mulher e ao recém-nascido no momento do parto e nascimento. Evidências brasileiras e internacionais apontam que modelos de atenção ao parto liderados ou fortemente apoiados pela enfermagem obstétrica estão associados a um maior uso de boas práticas, como alimentação, movimentação e métodos não farmacológicos para o alívio da dor durante o trabalho de parto, bem como ao aumento da probabilidade de parto vaginal espontâneo e menor utilização de intervenções, tais como a posição ginecológica, a pressão no fundo do útero e a episiotomia no momento do parto^(31,32)^. Por tais motivos, esses profissionais podem desempenhar papel estratégico tanto na identificação de barreiras ao início da amamentação - particularmente em situações de maior vulnerabilidade, como aquelas associadas à operação cesariana - quanto na oferta de orientações qualificadas, apoio emocional e incentivo às boas práticas desde a sala de parto.

### Limitações do estudo

O estudo foi realizado em uma instituição reconhecida pela qualidade assistencial e contou com a expressiva participação de 756 mulheres. No entanto, a seleção foi realizada por adesão voluntária, compondo uma amostra não probabilística, o que limita a generalização dos resultados e exige uma interpretação cautelosa por estar sujeita aos vieses comuns em estudos observacionais. Admite-se a possibilidade de viés de informação e de memória, uma vez que os dados foram obtidos por autorrelato, refletindo a percepção das participantes sobre as práticas assistenciais recebidas. Além disso, a coleta foi realizada por meio de formulário eletrônico, o que pode ter excluído pessoas com dificuldade de acesso à internet. Ainda, ressalta-se que não foram coletados dados sociodemográficos nem dados sobre complicações clínicas e obstétricas, o que impossibilitou a realização de análises de associação ajustadas para potenciais fatores de confusão.

### Contribuições para a Área

Este estudo reforça as evidências sobre os benefícios do critério global Cuidado Amigo da Mulher para o fortalecimento do aleitamento materno e o destaca como guia essencial para requalificar a assistência e reorientar o modelo obstétrico hegemônico, historicamente centrado no profissional, em direção a um cuidado centrado na mulher e baseado em evidências. Os achados demonstram que a adoção de tecnologias de cuidado que respeitam a fisiologia do parto e limitam o uso de intervenções desnecessárias favorece resultados positivos para a mulher, recém-nascido e equipe. Tais práticas estão diretamente relacionadas com a atuação de enfermeiras obstetras. Ao evidenciar a importância das práticas da IHAC na proteção, promoção e apoio ao aleitamento materno, o estudo também destaca o potencial estratégico da enfermagem na liderança de processos de avaliação e melhoria da qualidade assistencial em hospitais e maternidades, contribuindo para maior adesão e cumprimento das etapas recomendadas pela IHAC e pelas políticas públicas de atenção obstétrica e neonatal.

## CONCLUSÕES

As práticas de assistência ao parto, como garantir privacidade e um ambiente acolhedor, mostraram relação direta com o sucesso do aleitamento materno. O mesmo foi observado para as práticas que favorecem o aleitamento materno ao nascimento, entre elas o contato pele a pele, a identificação dos sinais de prontidão para mamar e o auxílio na colocação do bebê para mamar ainda no Centro Obstétrico. Esses achados reforçam a importância das políticas públicas como a IHAC e o critério global Cuidado Amigo da Mulher, que asseguram uma assistência mais humanizada, centrada na mulher e baseada em evidências. Para que tais princípios se consolidem na prática, é essencial que os profissionais de saúde estejam comprometidos e engajados na disseminação desse modelo de cuidado. Essa atuação contribui para a qualificação da assistência à mulher e ao recém-nascido, promove melhorias nos indicadores de saúde materno-infantil e favorece o desenvolvimento saudável das crianças desde os primeiros dias de vida.

## Data Availability

Os dados de pesquisa estão disponíveis em repositório: https://doi.org/10.48331/scielodata.OALXWW.
